# A smart device for non-invasive ADL estimation through multi-environmental sensor fusion

**DOI:** 10.1038/s41598-023-44436-5

**Published:** 2023-10-11

**Authors:** Homin Kang, Cheolhwan Lee, Soon Ju Kang

**Affiliations:** School of Electronic and Electrical Engineering, Kyungpook National University, Daegu, 41566 Republic of Korea

**Keywords:** Health care, Electrical and electronic engineering

## Abstract

This research paper introduces the Smart Plug Hub (SPH), a non-invasive system designed to accurately estimating a patient’s Activities of Daily Living (ADL). Traditional methods for measuring ADL include interviews, remote video systems, and wearable devices that track behavior. However, these approaches have limitations, such as patient memory dependency, privacy violations, and careless device management. To address these limitations, SPH utilizes sensor fusion to analyze time-series environmental signals and accurately estimate a patient’s ADL. We have effectively optimized the utilization of computing resources through the implementation of “device collaboration” in SPH to receive event data and segments portions of the time-series environmental signal. By segmenting the data into smaller segments, we extracted an analyzable dataset, which was processed by an edge device—SPH. We have conducted several experiments with the SPH, and our research has resulted in a significant 75% accuracy in the classification of patients’ kitchen ADLs and an 85% accuracy in the classification of toilet ADLs. These activities include actions such as eating activities in the kitchen and typical activities performed in the toilet. These findings have substantial implications for the progress of healthcare and patient care, highlighting the potential uses of the SPH technology in the monitoring and improvement of daily living activities.

## Introduction

Activities of daily living (ADL)^1,2^ are significant medical indicators that assess a patient’s ability to carry out routine activities in their daily life. ADL assessment is typically performed by evaluating a patient’s ability to carry out basic activities^3^ such as bathing, dressing, and using cooking appliances. Due to the direct observation of the patient’s behavior by a physician is the most effective way to assess ADL, because the number of doctors and their consultation time are insufficient, it is often impractical. Assessments are commonly carried out by means of interviews with the patient or their caregivers, video monitoring using closed-circuit television (CCTV) systems, or the utilization of wearable devices to observe the patient’s health condition^4^. However, it is important to acknowledge that each of these approaches has limitations, such as low reliability observed in interviews conducted with elderly patients who are suffering from degenerative brain diseases^5^. Additionally, there are concerns regarding privacy violations, as well as challenges associated with the proper usage of wearable devices. To facilitate the ongoing assessment of activities of daily living (ADL) without dependence on visual data, the utilization of a device capable of continuous measurement becomes necessary.

This paper introduces a novel edge device called the “Smart Plug Hub” (SPH). The SPH incorporates a range of sensors to detect environmental changes and uses the concepts of “Sensor Fusion” and “Device Collaboration” to infer human actions. Sensor Fusion is a data-processing technique that integrates and analyzes multiple sensor measurements to derive high-level inferences that cannot be achieved by a single sensor^6,7^. As human behavior leads to specific environmental changes, it becomes necessary to conduct a thorough analysis using multiple sensors in order to identify and understand the patterns associated with these changes. SPH has the capability to gather and analyze time-series signals obtained from a diverse range of environmental sensors, which exhibit variations in response to behavioral changes. Nevertheless, the process of analyzing time-series signals over a prolonged duration can prove to be inefficient due to the constant need for collecting and accumulating a substantial volume of signals. To tackle this concern, SPH employs the mechanism of “Device Collaboration”. A terminal device, referred to as a “device,” is utilized to convert raw sensor signals into abstracted signals. This device is capable of detecting triggering event data that leads to segmentation in the time-series environmental signals collected by the SPH. As SPH is equipped with the capability to support multiple communication protocols, it has the ability to receive event signals from a wide range of Internet of Things (IoT) devices within its proximity. Furthermore, it can effectively segment the area of sensed environmental signals based on various criteria. By conducting an analysis of the recorded environmental time-series signals and the received triggering event data, the SPH is able to infer the activities performed by the patient within the designated unit space at a given point in time. Moreover, it sends feedback signals to external devices, such as actuators attached to home appliances or other SPHs based on its deductions.

There have been previous studies on understanding human behavior from systems that integrate multiple sensors. Studies that employ sensor fusion techniques to integrate and analyze time-series signals from various sensors, including a smartphone’s built-in acceleration sensor, illumination sensor, microphone, and Wi-Fi scanning module, have been conducted. However, these studies have limitations as they do not consider environmental changes and do not utilize indirect data sources such as human voice detection^8^. In addition, studies that provide directions for predicting behavior using smartphone sensors do not use environmental sensor data and present sensor sampling with very short periods using acceleration sensors, which are difficult to use with low-power mobile devices^9^. Similar to the study discussed in this paper, a research investigation employing multiple environmental sensors has been conducted to gather data on environmental changes, acceleration, electromagnetic interference (EMI), and sound alterations. However, unlike the study mentioned, the collected data has not been analyzed at the device level^10^. The difference with this study is that it is difficult to provide real-time feedback to the patient at the device level, although the data is collected and compiled on an offline computer to perform machine learning. Unlike the aforementioned studies, the SPH proposed in this paper does not involve the transmission of sensor data to a remote server or cloud. Instead, it processes the data directly at the device level and offers real-time feedback to the patient.

The research contributions and objectives of this paper will be discussed in the following sections.

- We suggest a non-invasive ADL assessment approach through the use of IoT devices, avoiding the utilization of invasive wearable devices or recording devices that may cause privacy violations.
- The proposed system enhances the predictability of ADL by utilizing sensor fusion of various environmental time-series signals and improves accuracy. Furthermore, the system operates on an edge device, utilizing triggering event data to selectively process relevant data, thereby conserving computing resources.
- The SPH system reduces the risk of sensitive personal information breaches by performing data collection and analysis at the edge level, rather than transmitting raw data to external servers. Only inferred abstracted data is transmitted outside the network. Transmissions to the outside are restricted to specific purposes, such as analysis of ADL and notification to a doctor or caregiver, recognition of dangerous situations, operation of indoor actuators, or construction of a database for authorized personnel.
- In order to optimize the “Segmented signal” that can be obtained using Device Collaboration into the SPH system, we proposed a Predefined Weight Factor Mapping Algorithm in this paper. Similar to weights in the machine learning field, different predefined weights are applied depending on the appropriate space where ADL SPH are installed and the sensor type to estimate the ADL with highest probability.

A proposed Smart Plug Hub (SPH) edge device is designed to detect and predict residents’ activities of daily living (ADL) through the integration of multiple environmental time-series signals and collaboration with surrounding Iot devices. The paper begins with an introduction, followed by a review of related works. The conceptual properties of the proposed system are then discussed, along with a detailed design of the device. The article continues with the implementation and evaluation of the SPH, and concludes with a discussion of the study’s findings.

## Related works

Sensor fusion is a method used in various fields. Sensor fusion is an approach that uses several different sensors to capture information that is difficult to grasp with a single sensor^6^. In modern studies, sensor fusion is utilized in autonomous driving or autonomous robot control systems^11,12^. In autonomous driving, localization, motion estimation, data fusion, object recognition, path planning, action prediction, intercommunication, and obstacle avoidance are performed using sensors such as GPS, IMU (Inertial Measurement Unit), Radar, Camera, LiDAR, Ultrasonic, and V2X can be performed^13^. Such a sensor fusion mechanism and execution operation implementation are implemented on various operating systems and hardware platforms. In automatic robot control, contact switches, optical/magnetic encoders, infrared/LiDAR sensors, gyroscopes, and CCD/CMOS cameras for vision systems are used to overcome locomotion, navigation, and obstacle avoidance through sensor fusion. Various studies are also being conducted to monitor the conditions of indoor residents/patients^14^. The most widely used method is to analyze RSSI^14^, TOA^15^, TDOA^16^, CSI^17^ values using wearable terminals or smart devices, Predict the location of fixed nodes and moving residents/patients. Research is also being conducted to determine the location of residents in real time by analyzing images collected from each sensor node through a wireless visual sensor network and recognizing the indoor location without using a wearable terminals^18^. These studies can detect abnormal situations, such as when a resident does not move in a specific space for a long time or moves to a dangerous space, helping guardians or doctors to identify and treat behavioral patterns of residents.

The surrounding environment changes according to our behavioral patterns. Several studies have been conducted to measure and compare these changes using sensors. In particular, research is being actively conducted to detect the movement of body parts, such as arms and legs, and to deduce the actions that have been performed by attaching an inertial measurement unit(IMU)-based acceleration/gyro sensor to wearable terminals^19^. Research has also been conducted to record data from acceleration sensors inside smartphones that humans commonly carry and to identify stopping, sitting, and climbing stairs using deep learning models, not just using sensors attached to a human body^20^. In a study that presented a scenario in which a person identified a specific situation by attaching an inertial sensor to objects used in daily life without wearing an inertial sensor, sensor data were collected using fuzzy theory and used as machine learning data^21^. Similar to the research objectives presented in this paper, the disadvantage is that low-power implementation is disadvantageous when using acceleration sensors, and additional steps are needed to transfer raw data delivered by the sensor to the MQTT^22^ broker and post-processing by other computing devices. Unlike this study, the edge device proposed in this paper could receive post-processed data with the help of other external “Devices” and utilize computing power more efficiently. Studies are also ongoing which integrate acceleration sensor and environmental sensor (PIR) data to determine the exact location of the subject and predict the direction to move^23^. However, while wearable devices can identify various postures such as waking up, walking, and sitting in place, it is difficult to determine complex behaviors other than basic movements such as washing dishes and cooking food using accelerometer sensors. The “SPH”, an edge device proposed in this study, can predict what kind of behavior a person’s behavior was performed with only indirect data through communication between environmental sensor data and “Device” connected to SPH.

**Figure 1:**
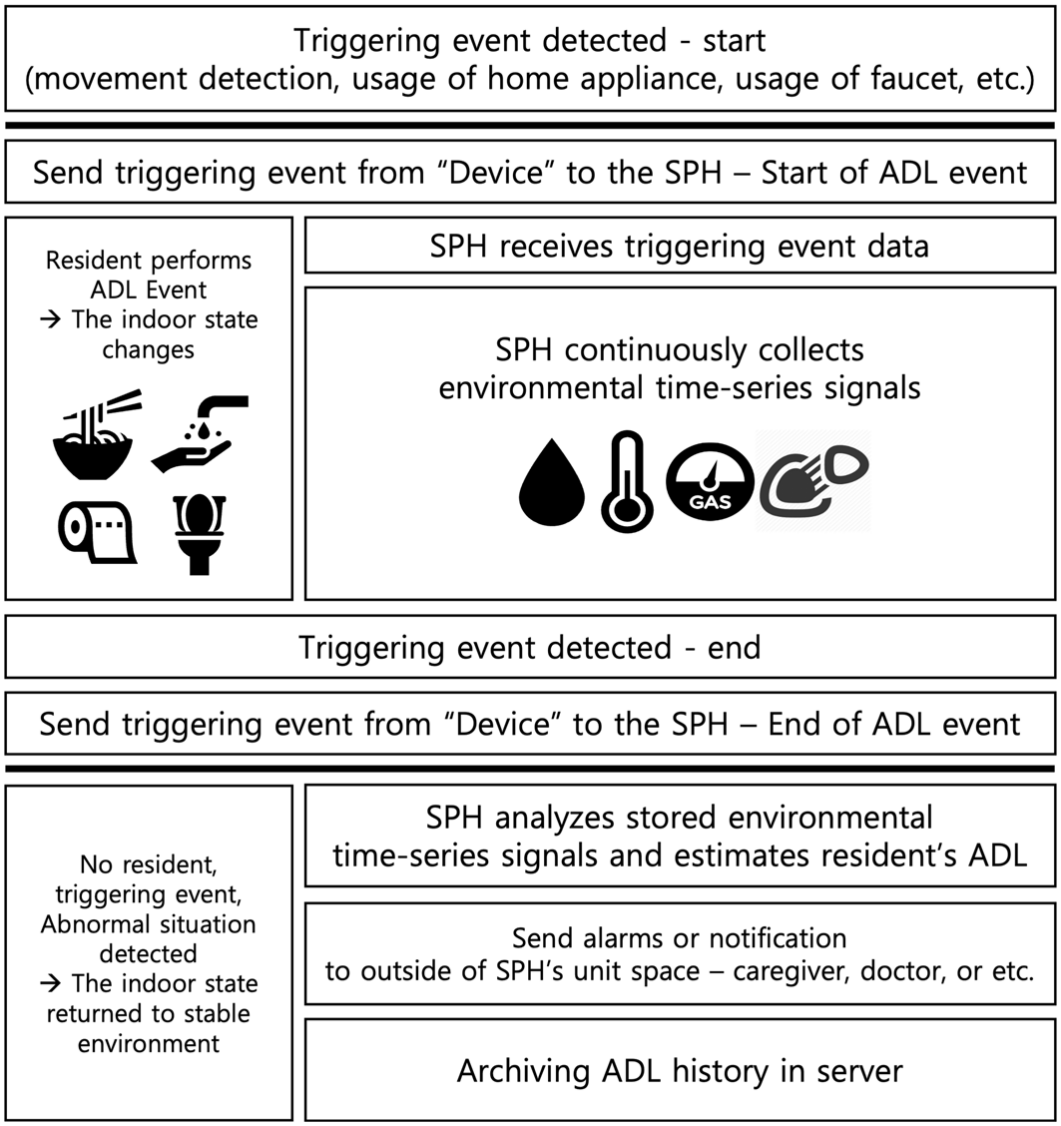
Overview of SPH System. The patient carries out an activity on the detection of the event. SPH predicts this activity by environmental time-series signals which was changed according to the patient’s ADL behavior.

## Conceptual properties of the proposed system

The proposed system’s conceptual properties are illustrated in Fig. 1. The SPH utilizes time-series environmental signals from various sensors and triggering event data from IoT “Devices” to predict the actions of patients. In this paper, “Devices” refers to all kinds of IoT devices that generate triggering event data that segment time-series signals collected by SPH (SPH supports a variety of wireless communication protocols because it segments based on triggering event data generated by the surrounding “device”). When the SPH receives triggering event data from the IoT “devices”, it segments the environmental time-series signals. After segmentation, the SPH analyzes the segment of the time-series signals to estimate emergency situations or the patient’s daily behavior. Depending on the estimation results, the operation of SPH is divided into one of the following: transmitting environmental time-series signals to an external server, transmitting estimation results to the external server or the other SPHs, or transmitting feedback or alarms to the caregivers of doctors. To implement the functions described above, SPH uses sensor fusion and device collaboration.

### Sensor fusion in SPH

**Figure 2:**
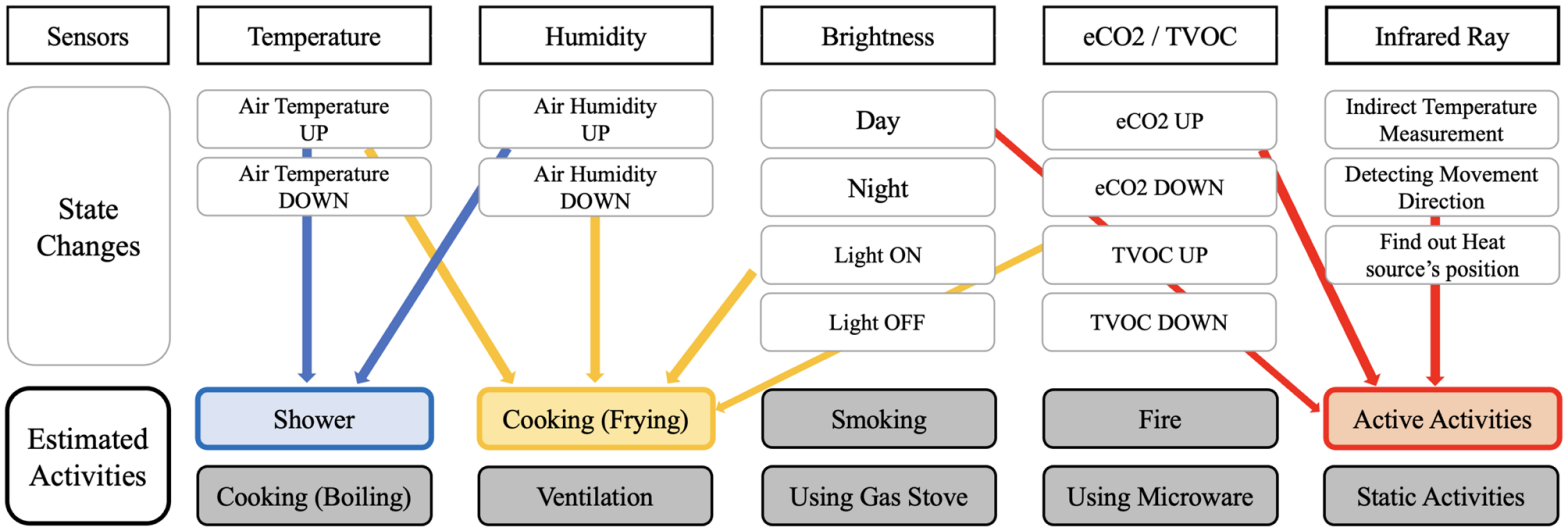
Mechanism and Advantage of Sensor Fusion. The gray features are the basic parameters of each sensor. With these features, colored features like taking a shower or cooking can be estimated through the sensor fusion mechanism.

A single sensor is limited to detecting only one type of state change that it is designed to measure. However, irrespective of the sensitivity of a single sensor, it may exhibit similar patterns for different behaviors of different individuals. For instance, differentiating between humid air from outside and artificially increased humidity inside, such as by a humidifier or boiling water in the kitchen, is challenging using only humidity. However, the possibility of distinguishing such activities increases when the temperature is analyzed concurrently for the same period, location, change value, and speed of the rate of temperature change. Combining signals from multiple sensors that measure different state changes to perform high-level situation determination, which cannot be established using a signal from a single sensor, is called “Sensor Fusion”^24^. As illustrated in Fig. 2, SPH incorporates various sensors for sensor fusion. The use of multiple sensors allows for the determination of more situations, and hence greater accuracy.

### Segmenting continuous-sensing time-series signals on the SPH

The SPH continuously collects environmental time-series signals and stores them in its internal memory or streams them into an external server. However, analyzing all the time-series signals at once on the edge device is not practical because of the large amount of processing signal. Instead, the objective is to determine what the person has done at a specific point in time. To analyze time-series signals, segmenting method is required to divide the signals into sections based on triggering event data^25^. The idea of dividing time-series signals into segmentation is also referred to as “Segmentation of time-series signals” in this paper. Whenever the SPH receives the triggering event data from the “devices”, it establishes the starting and ending points of the behavior and creates a segmented time-series signal collection to predict the patient’s action. Then, the SPH uses the segmented time-series signal as input for its prediction model.

**Figure 3:**
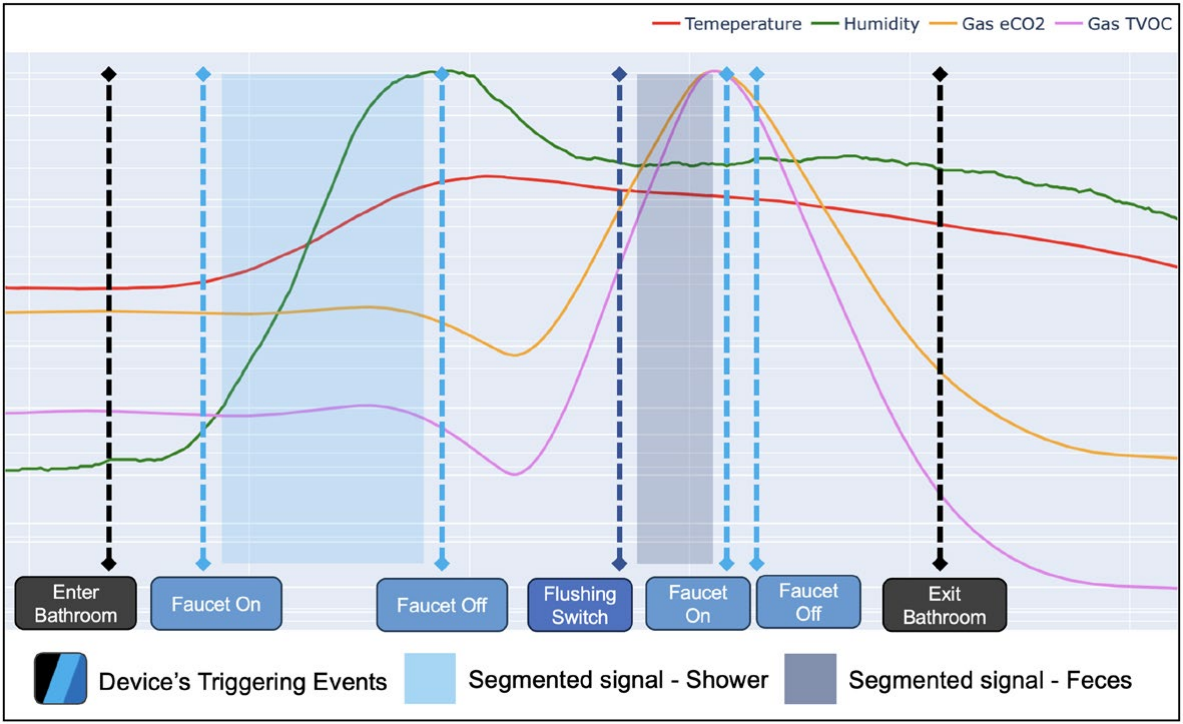
Segmenting continuously-sensing data. The situation-start point cannot be fixed by flows of time-series signal alone. Therefore, the SPH receives the triggering event data of the external “device” to begin the segmentation of time-series signals.

To implement this, it is necessary to assume that various types of IoT “devices” can be connected to the SPH. The SPH supports several types of connection methods, including Bluetooth^26^, IEEE802.11^27^, and 433 MHz Sub-1 GHz communication protocol, to connect multiple types of devices, which include not only self-developed devices but also commercial equipment. Figure 3 shows an example of the segmentation of time-series signals. Segmentation using only sensor data is a challenging task, but it can be made more reliable and easier by using other devices that generate triggering event data.

### Situation estimation and classification algorithm

**Figure 4:**
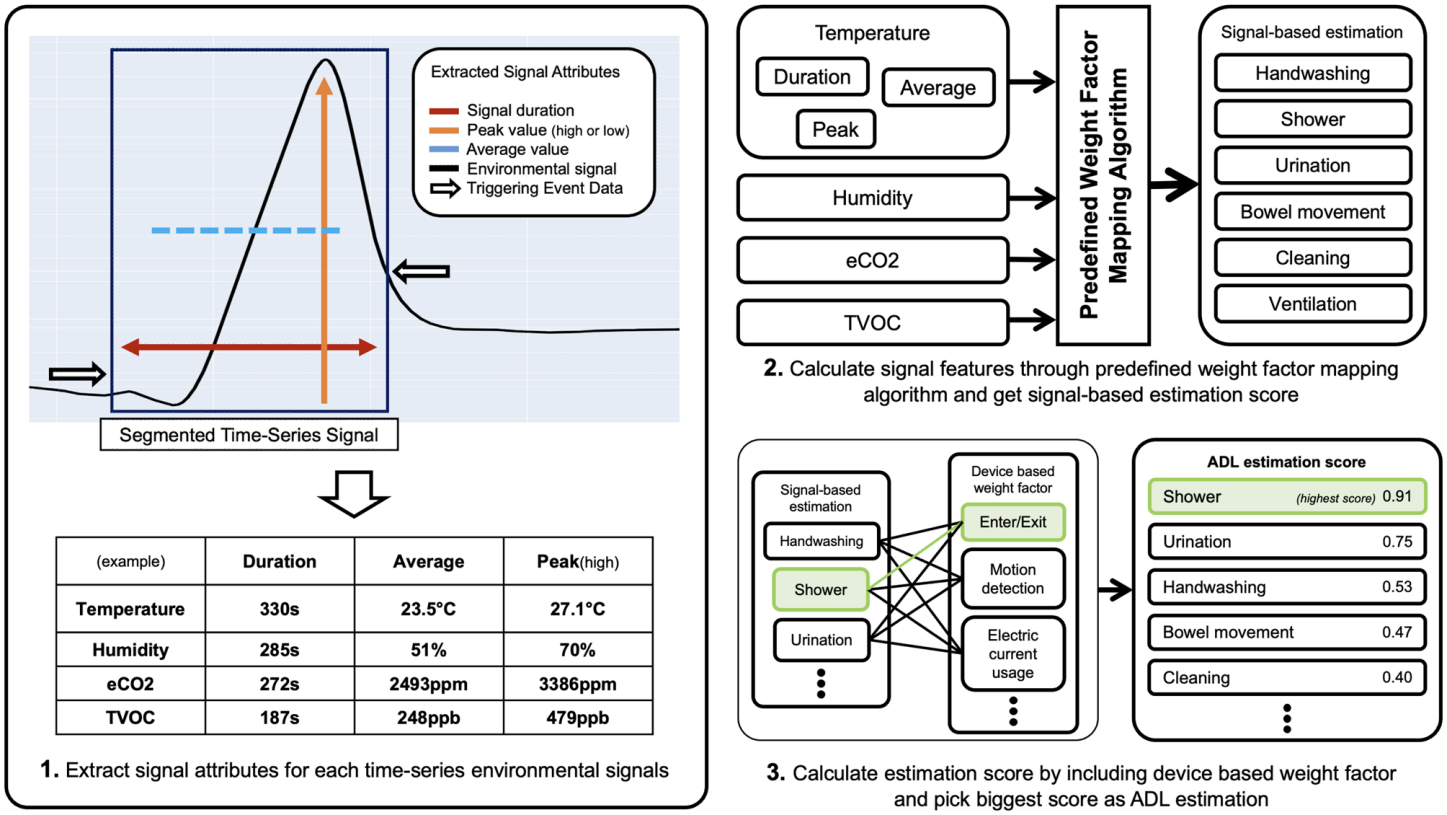
Signal reference analysis diagram. To estimate the ADL what the resident does, first, extract features from time-series signals, second, calculate.

The conventional approach for time-series classification has proven to be effective; however, it suffers from the drawback of limited real-time performance and high computational burden when executed in an edge device environment [citation needed]. Therefore, the present study introduces the predefined weight factor mapping algorithm (PWFMA), which demonstrates the ability to achieve real-time performance and low computational load. Figure 4 depicts the diagram outlining the procedure for conducting the PWFMA. All segmentation operations commence by receiving triggering event data from the device. Upon the reception of triggering event data, SPH initiates the process of extracting signal features from the environmental signals that were previously sensed. SPH continuously collects environmental signals and computes the duration, average, as well as the high or low peak value of each signal. These values represent information that can be dynamically updated in real-time during signal collection. They can be extracted without the need to store all segmented data within the SPH. When the subsequent data indicating the finish of signal segmentation is received, the duration, average, and peak values for each sensor type (specifically, temperature, humidity, eCO2, and TVOC values) can be retrieved from the SPH system. The features extracted through this methodology serve as input parameters for the PWFMA.

SPH is capable of extracting signal features and utilizing them to predict emergencies based on their duration and peak value before applying them to PWFMA. Both the act of cooking food in the kitchen and the presence of fire have the tendency to gradually elevate the temperature. However, there exists a significant disparity in terms of the magnitude and duration of temperature increase. Given the utilization of predefined data, it is possible to apply variations in duration, average, and peak values to SPH in the context of food cooking and fire. If the SPH extracts signal features that are excessively steep and abnormal, it will be classified as an emergency and will have the capability to notify external entities about the hazardous situation.

**Algorithm 1:**
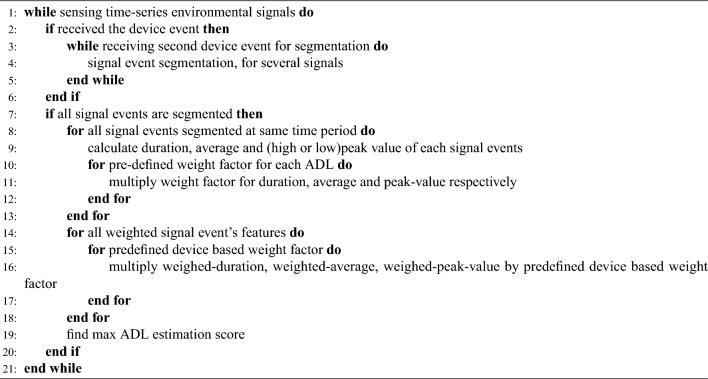
Predefined weight factor mapping algorithm.

The PWFMA is a computational method used to estimate the most probable Activities of Daily Living (ADL) performed by a patient. This algorithm applies various weight factors to the extracted signal features, depending on the specific ADL being analyzed [Algorithm 1]. Given that the sensor response sensitivity varies across different situations, it is necessary to establish varying levels of importance for each sensor based on the ADL. Essentially, when an individual enters a unit space and engages in an activity within the ADL category, the surrounding environmental signals inevitably change. Nevertheless, the outcome of the patient’s action can vary significantly, as some signals exhibit substantial changes while others remain relatively unaffected. For instance, during the act of showering in a bathroom, the signals recorded by the SPH device generally exhibit an upward trend. However, it is observed that the changes in humidity levels are more pronounced and occur at a faster rate compared to instances of urination or handwashing. In this particular scenario, in order to accurately estimate the occurrence of “having taken a shower” based on the corresponding signal, a more intensive analysis of the change in humidity is required compared to other signals. This is necessary to achieve a higher level of accuracy in the estimation process. The analysis and emphasis of sensors vary depending on the type of action, necessitating a consideration of different perspectives such as the time of action, magnitude of change, and rate of change. Therefore, the PWFMA assigns a unique weight factor to each ADL. By calculating the weight result based on the signal characteristics extracted from the segmented signal, the PWFMA estimates the ADL with the highest probability of being performed by indoor residents. The weight values in question are determined through empirical methods, which involve the combination of predefined reference data. Weight values should be assigned differently based on the unit space where SPH is installed, the ADL to be estimated, and the number and type of the sensors utilized.

After computing the estimated probability for each ADL based on the weighted signal features, the analysis also takes into account the device by which the signal was obtained. Signal division cannot always be accomplished solely through start/stop events originating from a single device. Given the existence of diverse device events, such as water usage, microwave usage, and refrigerator opening/closing, it becomes necessary to assign and take into account different weights based on these device-specific events. By incorporating the device base weight factor into the final calculation in SPH, taking into account the triggering event of each device, it is possible to derive the final estimated score for each ADL activity. The ADL estimation result can be determined by selecting the highest score among them. This estimation result can then be publicly announced or provided as feedback to residents within the indoor environment.

## Detailed design

### Hardware configuration

**Figure 5:**
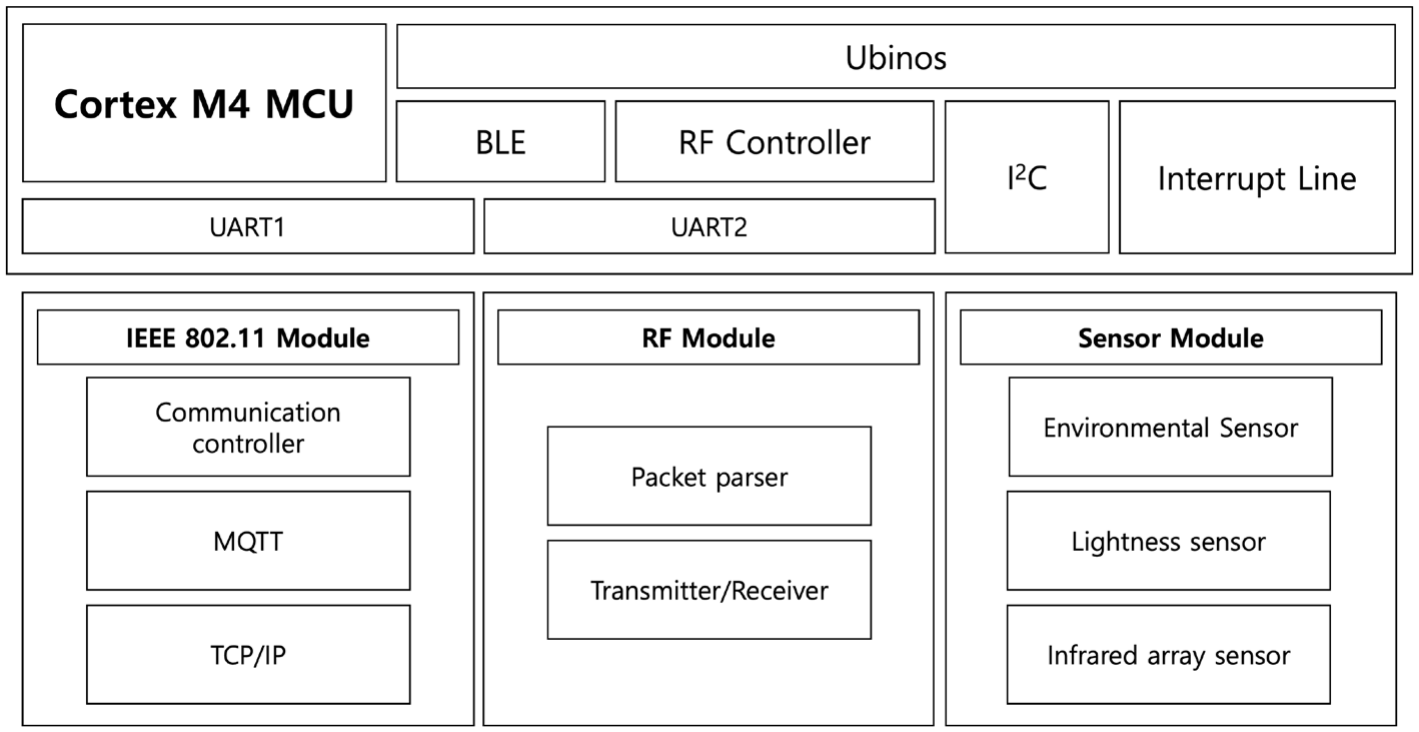
Hardware overview of Smart Plug Hub.

The system’s sensors and devices are presented in detail in Fig. 5. The SPH can manage up to 8 Bluetooth Low-Energy (BLE) peripheral devices that cover a small unit space. However, due to the limitations of the 2.4 GHz communication band and the BLE connection between edge devices for large amounts of data streaming^28^, only 3 to 5 peripheral connections can be reliably maintained. The SPH employees BLE, IEEE802.11, and 433 MHz RF wireless communication to interact with other SPHs or peripheral devices. The SPH differs from existing communication hubs since it comes with built-in sensors, allowing it to function in a stand-alone mode. A commercial wireless connection device can be integrated if the ID, authentication, and packet protocol information are available.

### Software configuration

**Figure 6:**
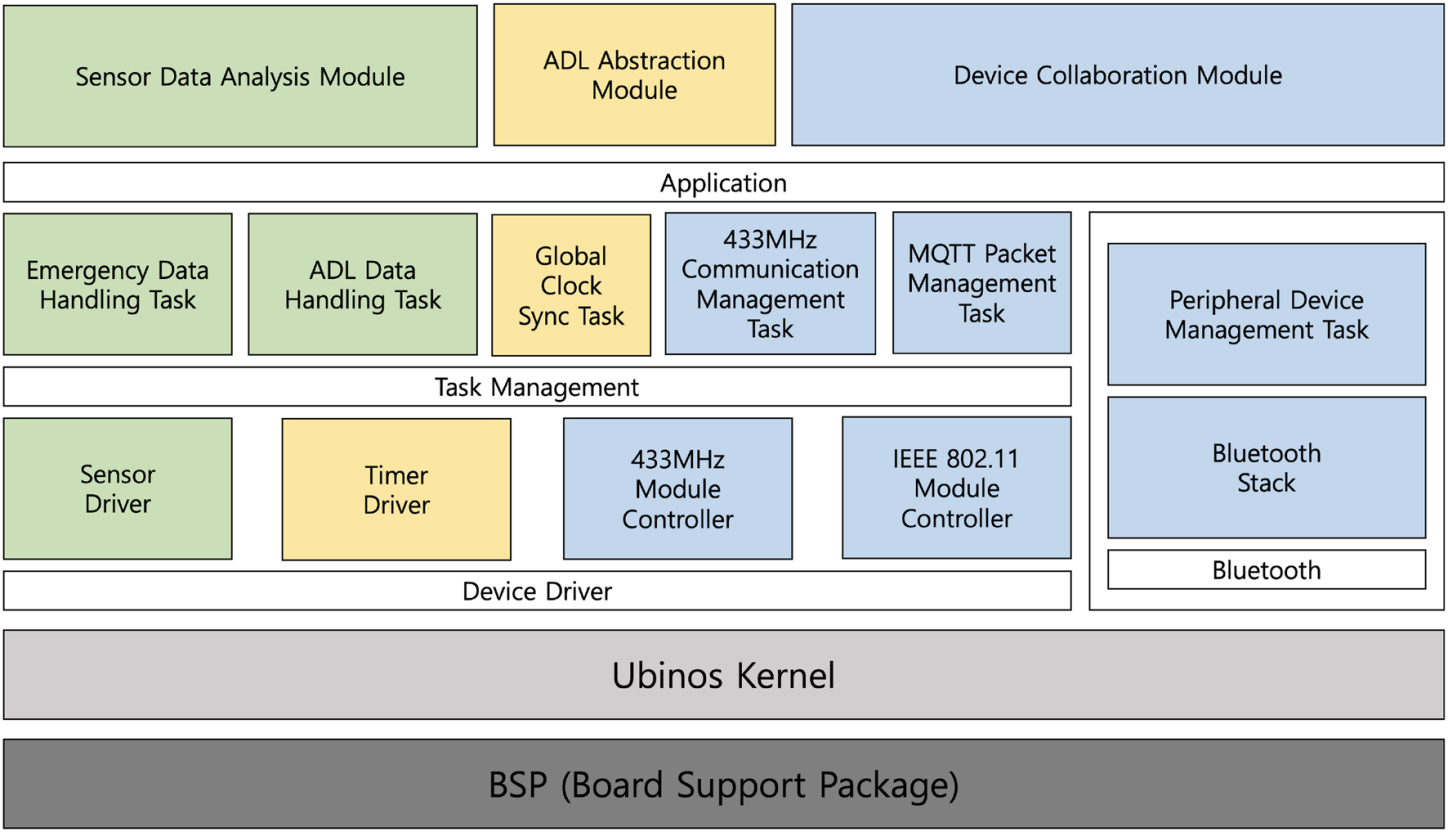
Software overview of smart Plug Hub.

Figure 6 depicts the software structure of the SPH, which operates on the Ubinos^29^ real-time operating system (RTOS). The SPH contains modules for communication with each sensor, which operate through a device driver and multitask for each function. The sensor’s time-series signal handler task delivers the sensor’s signal modified to the application that requested the time-series signals when specific events, such as timer events and triggering device data. The SPH can then transmit information to an external server via the Message Queuing Telemetry Transport Protocol^22^, transfer information to other devices of SPHs, or proceed with internal inference to generate additional data based on each event and application. Each operation is performed in an abstracted application layer, and the task management layer controls the detailed operations according to the multitasking technique of the Ubinos kernel.

### IoT devices for ADL identification

**Figure 7:**
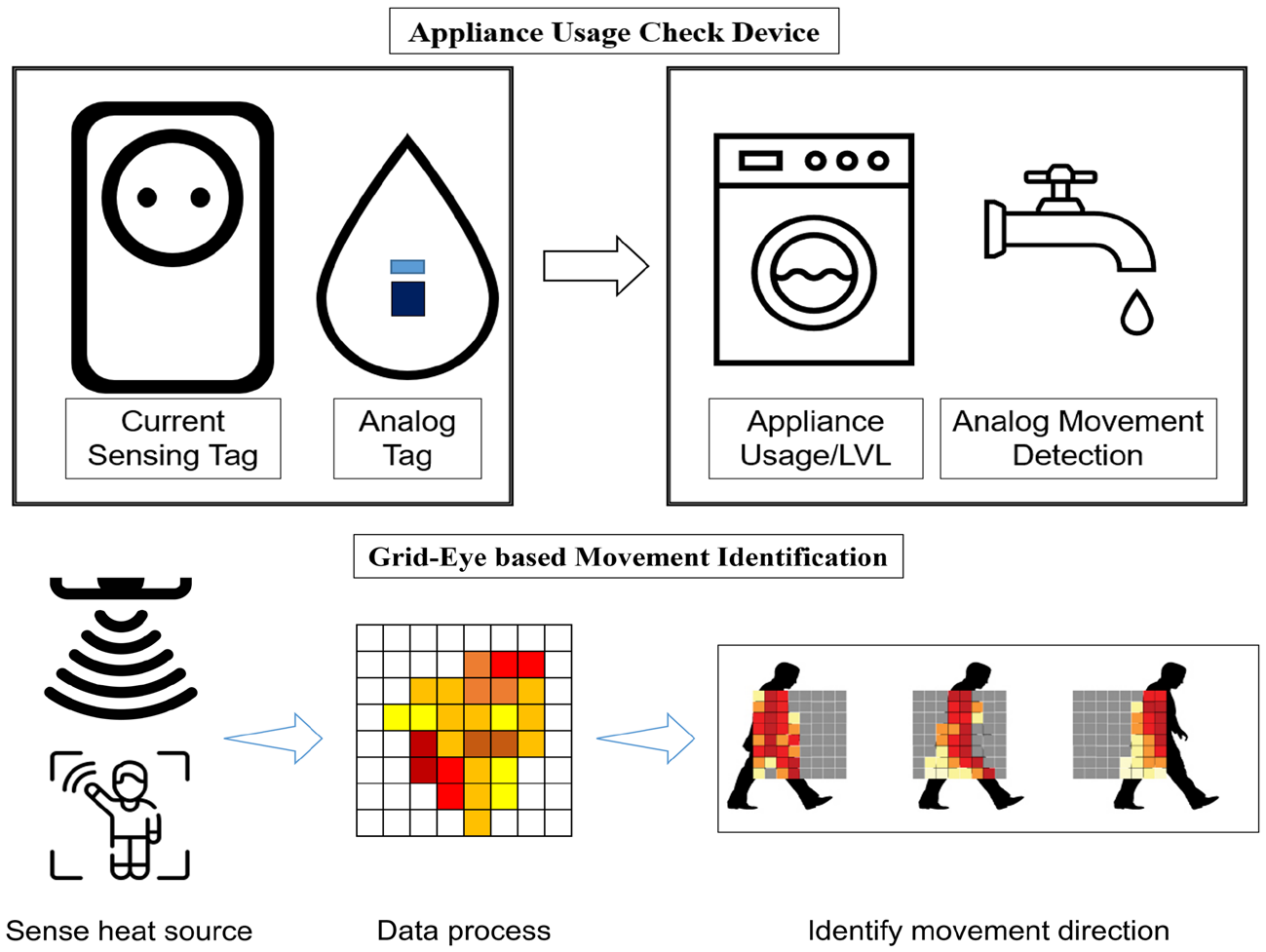
Concept of appliance usage check device and 2D infrared array sensor based movement identification.

This paper defines “device” as a tool that interprets raw signals gathered by a sensor that detects and measures simple state changes, converting it into abstracted data that can be easily comprehended by humans. For instance, a tilt measurement sensor can indicate the extent to which an object is inclined, but if the object is a lever that regulates the motor’s speed, it functions as an IoT “device” that can determine the current state of the motor. SPH collects triggering event data from various types of “devices” and uses them as a measure of data analysis to determine the situation. The primary content analyzed by SPH is a change in environmental sensor data collected according to behavioral changes. However, since analyzing data in all sections is inefficient and yields low accuracy, the time-series signal segmentation is performed by receiving triggering event data from the “device” linked to SPH. As explained above, SPH obtains data from different “devices” and conducts appropriate time-series signal segmentation. In this study, the “devices”, utilized for time-series signal segmentation, transmits triggering event data with a pre-processing sequence utilizing their own Micro Controller Unit (MCU). Figure 7 illustrates the “devices” that are used in this study, such as the 2D infrared array sensor^30^, current sensing tag^31^, analog analysis tag, etc., to analyze time-series signals in SPH.

Accurate measurement of certain activities, such as opening a door or using a faucet, is essential as it enables doctors to assess symptoms more efficiently based on ADL measurements. Thus, a specific home appliance’s usage is recorded to analyze the time-series signal accurately from the moment the appliance is turned off, and it can significantly simplify the analysis process. In this study, the use and mode of a home appliance were identified through the current sensing tag (CST), and an analog ADL tag identified appliances that cannot determine whether they are being used according to current intensity. These tags operate as “devices” connected to the SPH to facilitate signal processing by segmenting the time-series signals. The SPH supports various communication protocols, enabling the connection and processing of different “devices”. Furthermore, time-series signals collected by the SPH can be processed using “devices” with various applications such as door opening detection, PIR motion detection, and indoor light on/off detection using an ambient light sensor. The 2D infrared array sensor operated as a “device”, detects a heat source in a two-dimensional plane using an infrared temperature sensor, enabling the determination of the absolute temperature value in the direction of view. This sensor checks the direction in which the patient moves and generates triggering event data that is delivered to SPH to segment environmental time-series signals.

### Time-series signal handling and analysis

**Figure 8:**
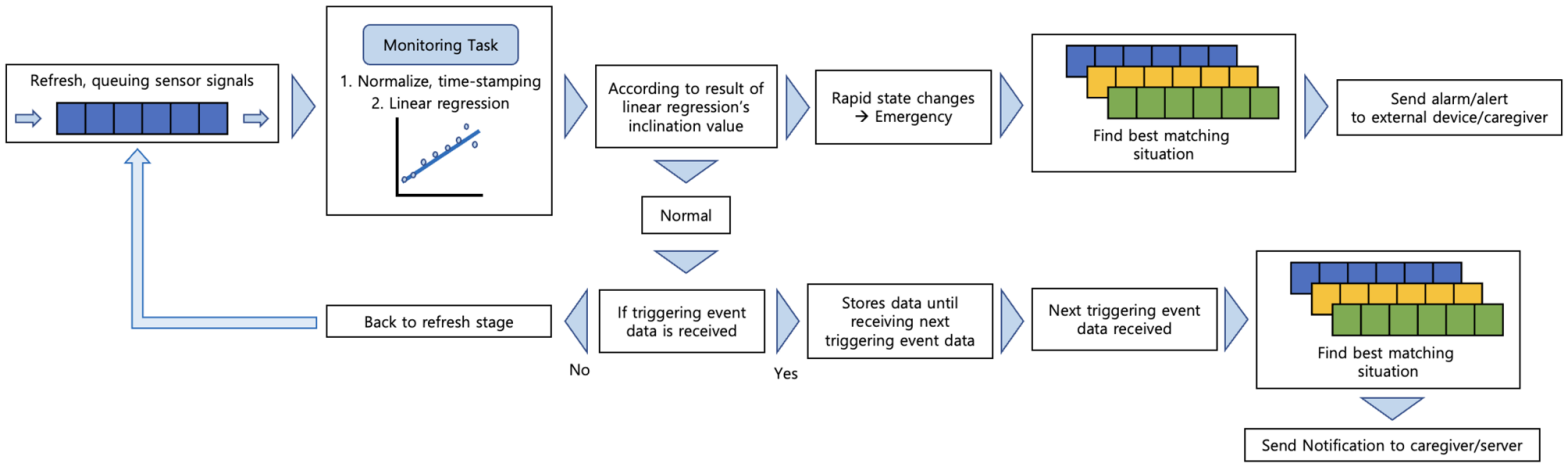
Detailed time-series signal handling procedure.

The SPH is designed to continuously collect environmental and noisy sound time-series signal, which is stored in its internal memory for a short period of time, with updates occurring at every sensing time. Monitoring and analyzing the time-series signal is performed by watching two memory regions: a short period of the start part of queue memory and all part of queue memory illustrated in Fig. 8. Initially, the SPH transforms the short period of internal time-series signals into normalized, timestamp-labeled signals for optimal analysis. Next, the SPH examines the pattern and determines the rate of signal change to identify whether an estimated situation is an emergency or a general situation using linear regression. If the state is normal—it means the absolute value of inclination of linear regression is small enough—the SPH resumes normal operation and continues to collect time-series environmental signals. If an emergency situation arises—it means the absolute value of the inclination of linear regression is too big—the SPH generate the triggering event data to notify other “devices” or other SPHs located in different unit space.

As ADL behaviors occur over different periods of time, ranging from short bathroom visits to longer meal times, the SPH requires time-series signals that span a longer period to estimate ADL. The length of the time-series signal collected for ADL estimation can be either variable or fixed, depending on the peripheral “device’s” triggering event signal. Variable-length time-series signal is determined by triggering event data such as emergency detection alarms from other SPHs, patient’s behavior detected by 2D infrared array sensors, or pre-processed triggering event data from wireless “devices” regarding the use of home appliances. As analyzing all data for an extended period is inefficient, the SPH stores sets of time-series signals for each ADL in advance. These “reference time-series signals” is stored in a remote server and can be applied by the SPH to estimate ADL. By evaluating the historical ADL data and sets of time-series signals estimated and transmitted by the SPH, human s can assess the performance of the SPH. When a developer selects a suitable ADL set from the server, they inform the SPH, which then compares the updated pre-defined ADL sets of time-series signals with newly collected signals.

## Implementation and evaluation

The performance evaluation was conducted with the aim of assessing three primary metrics: 1. The delay between triggering event and actual ADL activity, 2. The presence of any changes in the environmental time-series signals post-activity, and 3. Similar changes in environmental data with ADL.

### The delay between triggering event and actual ADL activity

**Figure 9:**
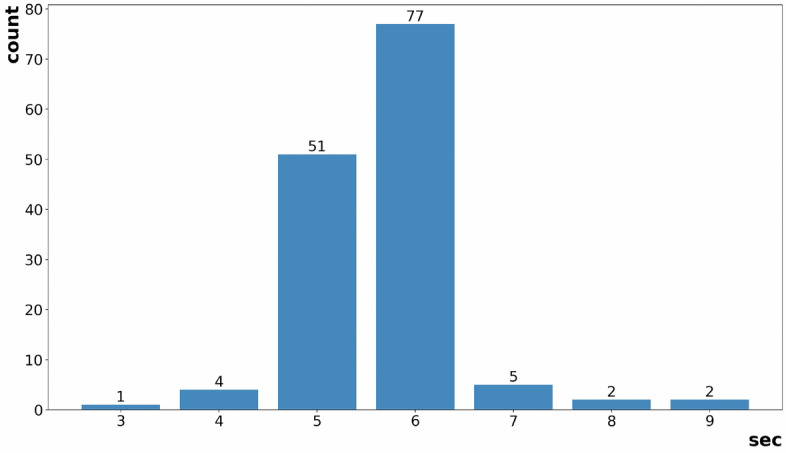
2D Infrared array sensor’s detection delay graph.

The estimation of Activities of Daily Living (ADL) in SPH is dependent on triggering event data. This data is generated by external devices or tasks that monitor environmental time-series signals and is subsequently utilized by the SPH. The 2D infrared array sensor is a highly effective means of capturing triggering event data, as it has the ability to detect the entry and exit of individuals within a defined area and monitor movement in front of specific household appliances. This capability greatly facilitates the segmentation of time-series signals. The detection delay of the SPH is characterized as the time interval between the detection of entry or exit by the 2D infrared array sensor and the execution of the corresponding ADL action.

**Table 1:** Delay comparison between ADL performing and entrance detection.

Average ADL assessment delay	2.11 s
Average entrance detection delay	5.67 s
Trial count	142 times

We conducted a series of experiments employing door-opening detection sensors directly connected to the host computer, and the SPH, connected to the host computer via Bluetooth. The SPH, positioned on the ceiling, utilizes an infrared 2D array sensor to detect the direction of movement of individuals passing beneath it. The SPH’s motion tracking feature is designed to identify movements in multiple directions, albeit with a slightly delayed response compared to the more immediate door opening detection. However, this feature allows for the detection of movements in various directions. Considering that the majority of Activities of Daily Living (ADL) involve entering or leaving a designated space, we utilized the “door opening” behavior as a triggering event to initiate specific ADLs in our experiments. The accurate measurement of the triggering event’s exact time is crucial, as the time-series signal is segmented based on this triggering event. The bar graph presented in Fig. 9 displays the time deviation between the detection time of the 2D infrared array sensor and the time of actual ADL performance (in this experiments, door opening). Also, the experiment summary is stated in Table 1. The analysis of the ADL behavior’s actual start time and the measured time indicated an average difference of approximately 2 s, using a standard based on typical adult males. The performance evaluation shows that the SPH receives, on average, triggering event data indicating the entry of a resident into the unit space after a duration of 5–6 s.

### The presence of any changes in the environmental data post-activity

The proposed edge device, known as SPH, has been specifically designed to gather and analyze time-series signals related to the environment, as well as triggering event data, from various devices located in residential settings. To validate our hypothesis, it was imperative to examine alterations in environmental data that are associated with ADL behavior. To this end, we conducted a long-term study by installing the SPH in a real-life residential setting.

**Figure 10:**
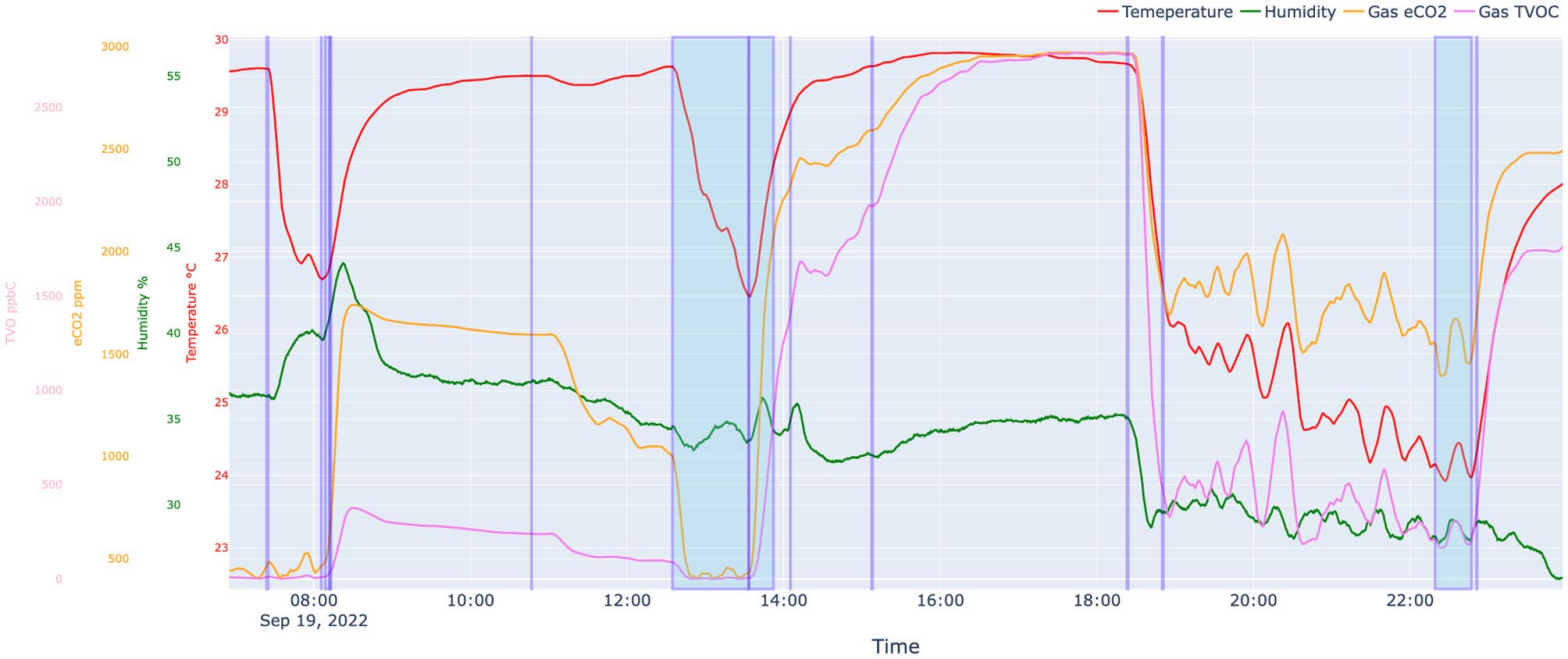
A graph displaying environmental data collected over a day the bathroom of an indoor resident. Each line graph is a time-series signal of environmental changes collected by multiple sensors, and the blue boxes and lines are the result of segmenting the significant behavior of a patient entering the bathroom.

A portion of time-series signals collected throughout a day is depicted in Fig. 10. The directional detection triggering event data from the 2D infrared array sensor is represented by blue boxes, while the line graphs illustrate the patterns in changes of environmental time-series signals. The data indicates that variations in environmental time-series signals exhibit greater speed and diversity subsequent to movements within the unit space, in comparison to other regions. This observation provides confirmation that the daily activities of patients result in environmental modifications that are conducive to their actions.

Merely observing changes in the environment based on daily behavior is inadequate for maintaining a comprehensive record of a patient’s ADL history. Therefore, it is imperative for the SPH system to possess the ability to identify and track environmental changes that align with each ADL performed by the patients. To evaluate its performance, we recorded the actions performed in each unit space as specific times daily and compared the results with the 2D infrared array sensor’s detection of access times and the occurrence of similar environmental changes for the same actions.

### Similar changes in environmental data with ADL

**Figure 11:**
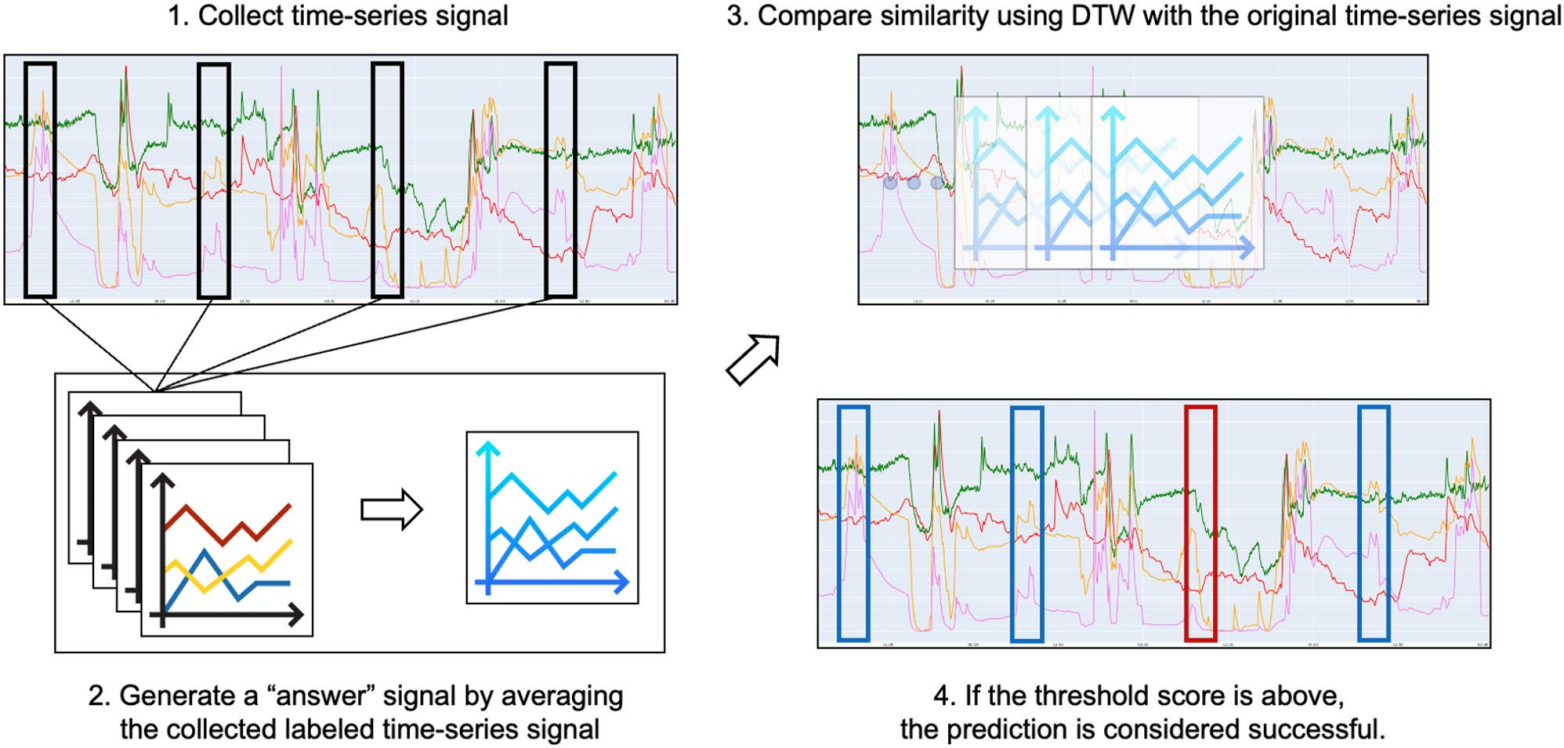
Examples of the relationship between the recorded ADL behaviors and the collected environmental time-series signals and triggering event data: movement.

In the present study, we conducted an investigation into the relationship between human behavior patterns and environmental changes. This was achieved by collecting time-series environmental signals using the SPH which was installed in a real-life residential setting. Therefore, data was collected from the patient’s living environment over a span of several weeks in order to investigate the potential correlation between human behavior patterns and patterns of environmental change. For a duration of several weeks, we conducted data collection of environmental signals in various areas such as the kitchen, bathroom, and unit space entry and exit. This was achieved through the utilization of 2D infrared array sensors. Additionally, we provided instructions to both patients and caregivers to accurately record the start and end times of their respective behaviors. We successfully acquired data that was annotated with various behaviors and confirmed that environmental modifications were indeed strongly correlated with human behavior, as hypothesized in this study. Additionally, the integration of sensor fusion proved to be beneficial in our analysis. Figure 11 depicts the evaluated method. The signals identified by the patient in the indoor setting were designated as “answer” signals, and a time-series signal was generated for each item. For instance, the breakfast meals were served a total of 20 times during a span of several weeks. We calculated the average of the 20 labeled signals and designated them as the “answer”. Subsequently, we assessed the similarity of the graph over the entire interval. The graph similarity was analyzed over the entire interval collected using the well-known method of Dynamic Time Wrapping (DTW)^32–34^.

Tables 2 and 3 present the results of the evaluation. False detection and miss detection in Tables 2 and 3 represent “SPH detected an ADL event but detected it differently from the actual behavior” and “SPH had an ADL event but did not detect it”, respectively. This phenomenon bears resemblance to the concepts of false positive and false negative in the field of machine learning.

**Table 2:** Kitchen event detection evaluation.

Target ADL event	Breakfast	Lunch	Dinner	Ventilation	Washing dishes	Activity
Correct detection	16	24	35	21	19	55
False detection	1	7	12	1	4	*
Miss detection	3	3	2	0	3	*
Accuracy	80%	70.6%	71.4%	95.4%	73%	*

To evaluate the SPH’s ability to accurately identifying specific behaviors, we conducted an analysis of the time-series signals obtained from the SPH installed in the kitchen and bathroom. During the study, patients were instructed to record the initiation and completion times of each targeted ADL behavior. Table 2 presents the results of the analysis, which revealed that events with predictable and rapid changes in execution times, such as breakfast and ventilation, were easily identified on the graph. However, the varying meal menus and meal durations of lunch, and the irregular meal menus and collective participation of most family members during dinner, made it challenging to accurately capture all the environmental changes caused by people. The “Activity” category in Table 2 denotes occurrences in which the 2D infrared array sensor detected movement, but the participant engaged in an action that was not the focus of the experiment.

**Table 3:** Bathroom event detection evaluation.

Target ADL event	Bowel movement	Urination	Shower	Ventilation	Activity
Correct detection	52	61	58	17	94
False detection	8	17	9	3	*
Miss detection	5	11	0	2	*
Accuracy	80%	68.5%	86.5%	77.3%	*

In addition, we analyzed the environmental time-series signals collected to observe the behaviors performed by the patient in bathroom, where changes in the environment such as using the toilet and taking a shower can be easily discerned. Table 3 presents the results of analysis, which revealed that activities with relatively long duration could be identified with a high degree of accuracy. The limited duration of urination led to negligible environmental alterations, posing challenges for the SPH sensor to effectively detect it in numerous cases. In the kitchen, the 2D infrared array sensor detected motion, indicating the occurrence of a triggering event. However, there were also events in the bathroom that were not targeted by the sensor for detection.

## Conclusion and future works

This study proposes the design and evaluation of the Smart Plug Hub (SPH), an edge device that collects and analyzes environmental time-series signals and triggering event data from surrounding IoT “devices” in residential environments. The SPH can detect and estimate the patient’s Activities of Daily Living (ADL) through the integration of multiple environmental time-series signals and collaboration with IoT “devices”. The advances over previous research that we would like to highlight in this paper are that we: (1) correlated environmental data with ADL behavior, and (2) used device-triggered event data to segment the time series signal. It has been established through numerous studies and human experience that environmental changes are influenced by human behavior. However, it is important to note that there are distinct environmental changes associated with each behavioral pattern. In addition, a significant strength of this paper is the segmentation of the time-series signal using triggering event data from the “device” that identifies specific human behaviors. This segmentation allows for the identification of patterns in edge-level devices. The study conducted a long-term experiment in a real-life residential setting to observe changes in environmental time-series signals that are correlated with ADL behavior. These signals are critical for maintaining a comprehensive record of a patient’s ADL history. The results demonstrate a clear association between resident movement and environmental changes, as well as the SPH’s ability to detect patterns of environmental change that correspond to each ADL action performed by the patient.

However, the proposed SPH device uses wired power and does not take lower power consumption into consideration. In future research, the authors plan to develop mobile SPH devices with low power consumption by reducing the number of sensors and devices connected using wireless communication protocols. The collected set fo time-series signals will be used to build machine/deep learning models and compare their performance with the algorithms applied in this study. These developments will enable more efficient and accurate ADL evaluation and improve the use of SPH devices in healthcare settings.

## Data Availability

The data collected during and/or analyzed during this study is available from the corresponding author if the patients, involved in this study, allows.
